# Distal lung epithelial progenitor cell function declines with age

**DOI:** 10.1038/s41598-020-66966-y

**Published:** 2020-06-26

**Authors:** Julie K. Watson, Philip Sanders, Rebecca Dunmore, Guglielmo Rosignoli, Yvon Julé, Emma L. Rawlins, Tomas Mustelin, Richard May, Deborah Clarke, Donna K. Finch

**Affiliations:** 1 Research and Early Development, Respiratory & Immunology, BioPharmaceuticals R&D, AstraZeneca, Cambridge, UK; 2 Biocellvia, 10 rue Grignan, Marseille, 13001 France; 3 Gurdon Institute, University of Cambridge, Tennis Court Rd., Cambridge, CB2 1QN UK

**Keywords:** Ageing, Stem-cell differentiation

## Abstract

Tissue stem cell exhaustion is a key hallmark of aging, and in this study, we characterised its manifestation in the distal lung. We compared the lungs of 3- and 22-month old mice. We examined the gross morphological changes in these lungs, the density and function of epithelial progenitor populations and the epithelial gene expression profile. Bronchioles became smaller in their cross-sectional area and diameter. Using long-term EdU incorporation analysis and immunohistochemistry, we found that bronchiolar cell density remained stable with aging, but inferred rates of bronchiolar club progenitor cell self-renewal and differentiation were reduced, indicative of an overall slowdown in cellular turnover. Alveolar Type II progenitor cell density and self-renewal were maintained per unit tissue area with aging, but rates of inferred differentiation into Type I cells, and indeed overall density of Type I cells was reduced. Microarray analysis revealed age-related changes in multiple genes, including some with roles in proliferation and differentiation, and in IGF and TGFβ signalling pathways. By characterising how lung stem cell dynamics change with aging, this study will elucidate how they contribute to age-related loss of pulmonary function, and pathogenesis of common age-related pulmonary diseases.

## Introduction

Tissue stem cell exhaustion is a key hallmark of aging^1^, but has not been fully investigated in the lung. It is important to characterise how lung stem cell dynamics change with aging, and how they contribute to age-related loss of pulmonary function. Chronic obstructive pulmonary disease (COPD), idiopathic pulmonary fibrosis (IPF) and lung cancer typically onset in old age, and it is thought that aging contributes to their pathogenesis. A detailed understanding of the changes that occur in healthy aging will provide a baseline for understanding the further changes that occur during the development of common age-related pulmonary diseases.

Healthy lung aging is characterised by complex multiscale changes in the physical structure of tissue and in its cellular and molecular composition. There is a stiffening of the chest wall, a decrease in respiratory system resistance, and an increase in dynamic compliance and hysteresis^2–5^. Lung tissue typically becomes less able to regenerate with aging. This has been described in well-defined animal models where there is an absence of the confounding variables typically found in the human population. For example, in mice, there is a progressive loss in regenerative capacity in response to insults such as pneumonectomy, influenza, or E. coli lipopolysaccharide^6–11^.

Specific changes in defined progenitor cell populations likely underlie the changes in structure and function of the lung. Previous work showed that in the aging mouse upper airways, the number of basal epithelial progenitor cells is decreased, and that this is accompanied by a reduction in overall epithelial cell density. Additionally, gland-like structures bud off from the surface epithelium^12,13^. In the distal lung, there is an enlargement of the airspaces, however alterations in progenitor number and function in the distal lung has been less well explored^4,5,14^.

The aging process is thought to be directly related to the pathogenesis of IPF and COPD in particular, as epithelial senescence is a key driver of these diseases. Notably, studies of familial IPF have identified inherited mutations in telomerase (TR) and telomerase reverse transcriptase (TERT) that cause IPF^15^, and a GWAS study has identified common SNPs in TERT that are risk factors for IPF development^16^. In mouse models, disruption of telomerase activity specifically in alveolar Type II epithelial progenitors drove development of pulmonary fibrosis^17^. This highlights the importance of epithelial senescence in driving pathogenesis, although fibroblast senescence may also play a role^18^. Suicide gene-mediated ablation of senescent cells in bleomycin-treated mice improved pulmonary function, and treatment of IPF fibroblasts or epithelial cells with a senolytic cocktail *in vitro* cleared senescent cells^19^. Telomere shortening is also observed in COPD endothelial progenitor cells and leukocytes^20,21^, as is increased DNA damage response at telomeres in COPD airway epithelial cells^22^.

In this study, we compare the distal lungs of 3- and 22-month old mice. We examine the gross morphological changes in these lungs, the number and function of epithelial progenitor populations and the epithelial gene expression profile. We find that bronchioles become smaller in their cross-sectional area and diameter. We examine density and infer fate decisions of bronchiolar club and alveolar Type II progenitor cells by means of long-term EdU incorporation analysis and immunohistochemistry. We find that overall bronchiolar cell density remains stable with aging. We infer that overall rates of club cell self-renewal and differentiation are however reduced, indicative of an overall slowdown in cellular turnover. We find that Type II cell density and self-renewal are maintained with aging. We find that overall density of Type I cells is reduced, and infer that this is due to reduced Type II to Type I cell differentiation. We examine age related changes in lung epithelial gene expression profile by microarray analysis, and find changes in multiple genes, notably including some with roles in proliferation and differentiation, and in several signalling pathways, including the IGF and TGFβ pathways.

## Results

### Study design

To study the aging lung, we compared cohorts of 3- and 22-month old C57/Bl6J mice. Mice aged 6–8 weeks old are commonly taken to be adults, but we used slightly older 3-month old mice as our baseline group, to eliminate any effects associated with maturation. Calculations of median C57/Bl6 lifespan range from 18–29 months for females and 21–31 months for males^23^. Variation may be due to different diets or exercise levels. Under our standard conditions, we found it possible to consistently age mixed-gender cohorts to 22 months with negligible mortality.

### Aged lung tissue is less dense, with bronchioles that have a smaller cross-sectional area

**Figure 1:**
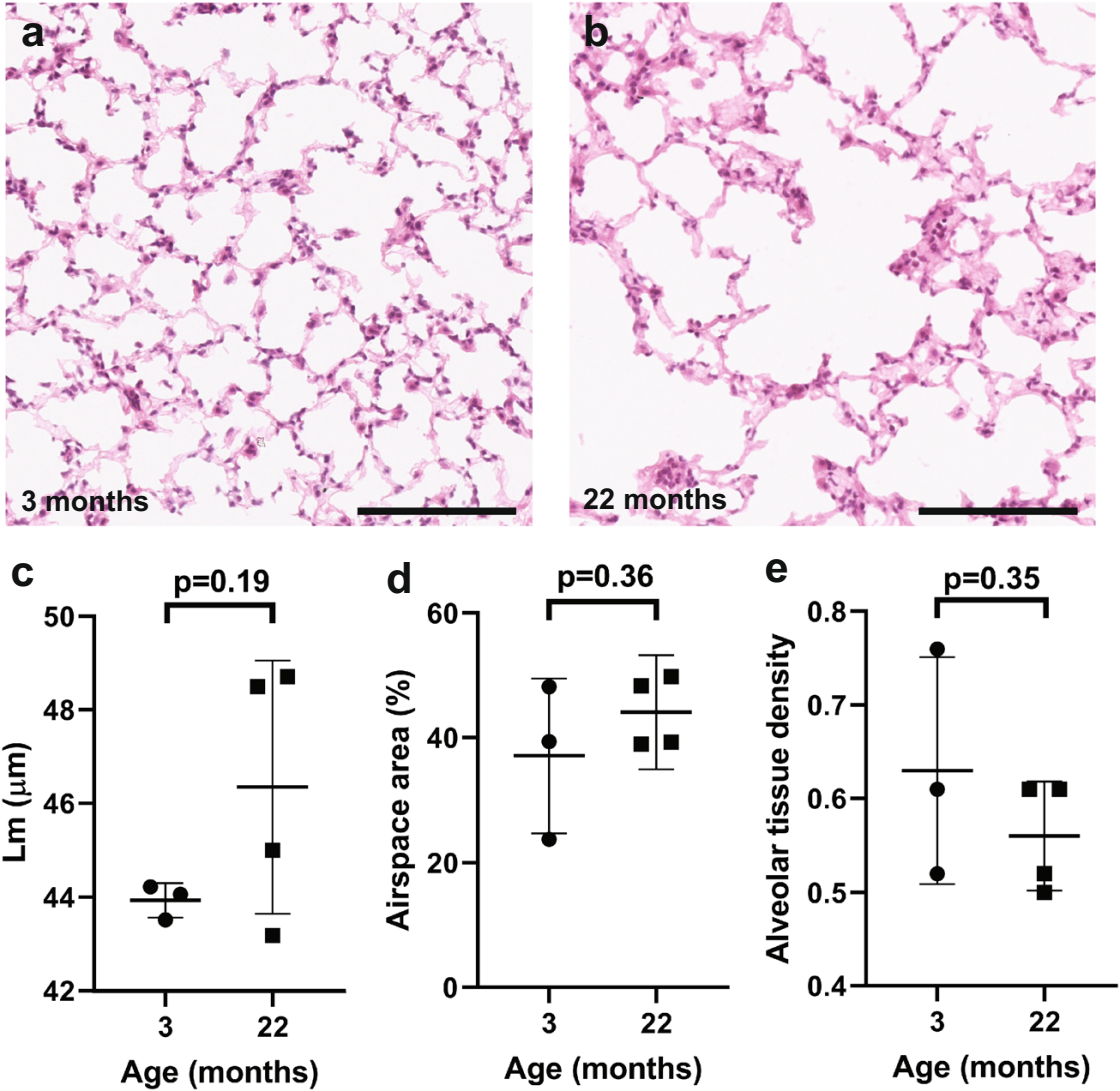
Lung parenchymal density at different ages. Representative 40x images of H&E stained lung parenchyma from (**a**) 3 month old and (**b**) 22 month old mice. Scale bars are 150 µm. Dotplots depicting (**c**) mean linear intercept (Lm), (**d**) airspace area (>2 images analysed per mouse) and (**e**) alveolar tissue density (>2 images analysed per mouse). Circles represent 3 month old mice, and squares represent 22 month old mice. Error bars are standard deviations. P-values refer to two-tailed T-test results.

Gross morphology was determined by H&E staining of multiple slides from serially sectioned lungs, and imaging of whole lung sections. Automated image quantification was carried out by Biocellvia (Marseille), using their validated proprietary software programs^24,25^ (Supp Fig. 1). Biocellvia automated image analysis eliminates investigator bias, and provides a high level of accuracy and reliability. Previous studies have identified airspace enlargement with aging^4,5,14^. We could not confirm this, although we found a trend towards airspace enlargement with aging. Mean Lm value was 44 µm ± 0.4 µm at 3 months and 46 µm ± 3 µm at 22 months (p = 0.19, Fig. 1a–c). Mean airspace area (the percentage area covered by airspace, rather than tissue) was 37% ± 12% at 3 months and 44% ± 6% at 22 months (p = 0.36, Fig. 1a,b,d, Supp Fig. 1e). The mean alveolar tissue density was 0.63 ± 0.12 at 3 months and 0.56 ± 0.06 at 22 months (p = 0.35, Fig. 1a,b,e Supp Fig. 1e).

**Figure 2:**
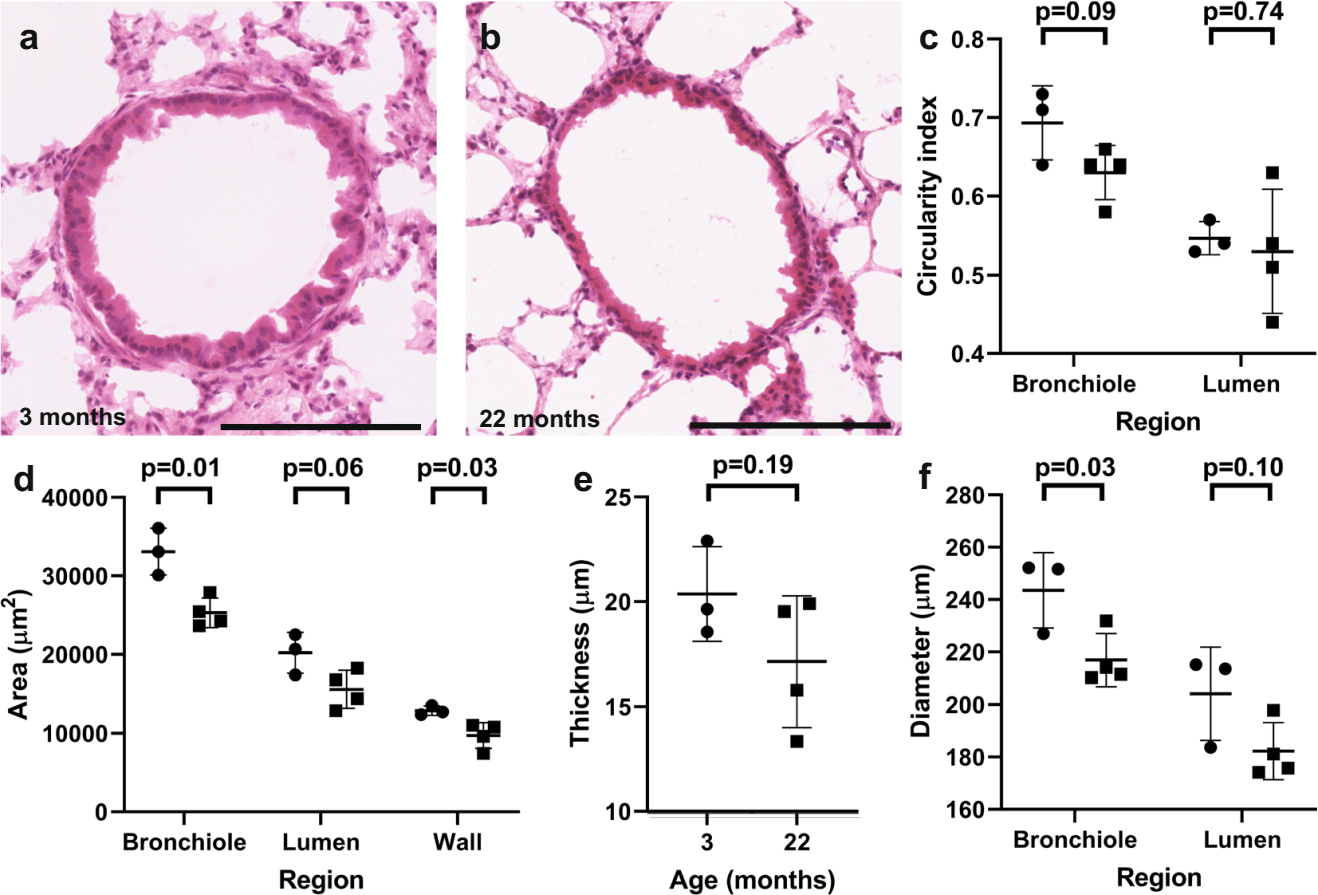
Bronchioles become smaller in their cross-sectional area and diameter with age. Representative 40x images of H&E-stained distal lung bronchioles from (**a**) 3 month old and (**b**) 22 month old mice. Scale bars are 150 µm. Dotplots depicting (**c**) Circularity of the whole bronchiole and the lumen, (**d**) Area of the whole bronchiole, the bronchiolar lumen and the bronchiolar wall, (**e**) Thickness of the bronchiolar wall, (**f**) Diameter of the whole bronchiole and bronchiolar lumen. Circles represent 3 month old mice, and squares represent 22 month old mice. >7 images were analysed per mouse. Error bars are standard deviations. P-values refer to two-tailed T-test results.

There was no change in circularity of whole bronchioles (p = 0.09) and that of their lumens (p = 0.74, Fig. 2a-c). Total cross-sectional area of bronchioles was reduced by 24% with aging, from 33096 µm^2^ ± 2976 µm^2^ at 3 months to 25308 µm^2^ ± 1875 µm^2^ at 22 months (p = 0.01, Fig. 2a,b,d, Supp Fig. 1a–d). This was due to a 25% lower bronchiolar wall area (12888 µm^2^ ± 594 µm^2^ at 3 months, 9271 µm^2^ ± 1636 µm^2^ at 22 months, p = 0.03), and a trend towards a 23% lower lumen area (20207 µm^2^ ± 2595 µm^2^ at 3 months, 15587 µm^2^ ± 2405 µm^2^ at 22 months, p = 0.06). The altered bronchiolar morphology was also reflected in a 11% thinning of bronchioles with aging, from 243 µm ± 14 µm diameter at 3 months to 217 µm ± 10 µm at 22 months (p = 0.03, Fig. 2a,b,f, Supp Fig. 1a–d). Lumen diameter was 204 µm ± 18 µm at 3 months and 182 µm ± 11 µm at 22 months (p = 0.10, Fig. 2a,b,f), whilst bronchiolar wall diameter was 20.4 µm ± 2.3 µm at 3 months and 17.1 µm ± 3.1 µm at 22 months, p = 0.19, Fig. 2a,b,e, Supp Fig. 1a–d). This is to our knowledge the first demonstration of decreased bronchiolar cross-sectional area and diameter with aging.

### Proliferative activities of alveolar Type II cells and bronchiolar club cells change with aging

To assay progenitor cell proliferation, mice were administered EdU ad libitum in their drinking water for 14 days. Administration of a nucleotide analogue in this manner over a prolonged period is preferred for aging studies^26^ to ensure that a high baseline level of dividing cells is labelled in young mice, such that subtle age-related changes in proliferation rates are detectable. Progenitor cell proliferation involves balanced self-renewal and differentiation. Therefore, over the 14 day period of the experiment, EdU would be incorporated into dividing progenitors and carried into their progeny (progenitors produced by self-renewal and terminally differentiated cells produced by differentiation). Immunohistochemical staining of multiple slides from serially sectioned lungs was carried out to identify progenitors and terminally differentiated cells that were, and were not, EdU+. The percentage of EdU+ progenitors was therefore used to infer self-renewal capacity, and the percentage of EdU+ terminally differentiated cells was used to infer differentiation capacity of their upstream progenitors.

**Figure 3:**
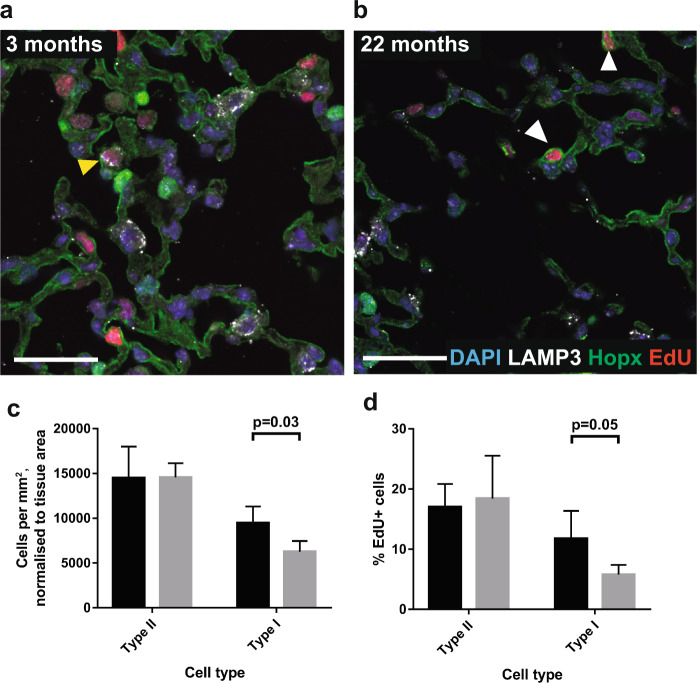
Alveolar Type II progenitor density and proliferation is maintained with age, but emergence of EdU+ Type I cells is decreased. 40x images of lung alveoli from (**a**) 3 month old and (**b**) 22 month old mice treated with EdU ad libitum in drinking water for 14 days, stained for DAPI (blue), LAMP3 (white, Type II cells), Hopx (green, Type I cells), EdU (red, cells that have proliferated). Scale bars are 30 µm. Yellow arrowhead indicates EdU+ Type II cell, white arrowheads indicate EdU+ Type I cells. (**c**) Histogram depicting density of Type II and Type I cells per mm^2^ of alveolar tissue area. Note that data has been normalised to the alveolar tissue density values shown in 1c, to eliminate effects of decreased density with age. (**d**) Histogram depicting percentage of Type II and Type I cells that have incorporated EdU, indicating that they have undergone proliferation. >950 cells were counted per mouse, and 4 mice were in each age group. Black bars represent 3 month old mice, and grey bars represent 22 month old mice. Error bars are standard deviations, and p-values refer to two-tailed T-test results.

In the alveolar region, the number of Lamp3+ Type II progenitor cells and terminally differentiated Hopx+ Type I cells was counted and normalised to tissue density (tissue density measurements based on data in Fig. 1c). The normalised data therefore reflect cellular density within a unit area of the alveolar walls. Importantly, Lamp3 co-localized with another Type II cell marker, Sftpc, whilst Hopx co-localized with another Type I cell marker, T1α, in young and old lungs, demonstrating that these markers are sufficiently specific for our analysis (Supp Fig. 2). Density of Type II cells remained constant (14,490 ± 3519/mm^2^ at 3 months, 14,559 ± 6279/mm^2^ at 22 months) whereas density of Type I cells fell by 34% (9453 ± 1863/mm^2^ at 3 months, 6279 ± 1173/mm^2^ at 22 months, p = 0.03, Fig. 3a-c).

The proportion of EdU+ Type II cells remained constant over the lifespan, indicating a maintenance of Type II cell self-renewal capacity (17.0% ± 3.8% at 3 months, 18.4% ± 7.1% at 22 months). On the other hand, the proportion of EdU+ Type I cells fell from 11.8% ± 4.6% at 3 months of age to 5.8% ± 1.6% at 22 months of age. As Type I cells have been reported to be terminally differentiated at homeostasis and derived from Type II cells^14,27,28^, we infer a halving of Type I cell production from Type II cells over the experimental period (Fig. 3a,b,d). An inferred reduction in Type II-Type I cell differentiation rate would be sufficient to drive the reduction in Type I cell density seen in the aged lung. Our data is therefore consistent with a scenario in which there is no increase in Type I cell death rates with aging, although we cannot exclude this possibility.

**Figure 4:**
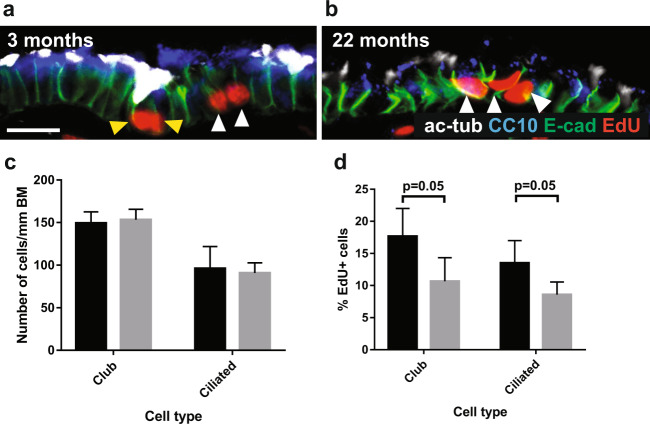
Density of bronchiolar club and ciliated cells is maintained with aging, but proliferation rates of club cells decrease. 40x images of bronchioles from (**a**) 3 month old (n = 4) and (**b**) 22 month old (n = 4) mice treated with EdU ad libitum in drinking water for 14 days, stained for CC10 (blue), acetylated tubulin (white, ciliated cells), E-cadherin (green, epithelial cell adherence junctions), EdU (red, cells that have proliferated). Scale bars are 30 µm. Yellow arrowheads indicate EdU+ ciliated cells, white arrowheads indicate EdU+ club cells. Histograms depicting (**c**) density of club and ciliated cells per mm bronchiolar basement membrane, and (**d**) Percentage of club and ciliated cells that have incorporated EdU, indicating that they have undergone proliferation. >200 cells were counted per mouse, and 4 mice were in each age group. Black bars represent 3 month old mice, and grey bars represent 22 month old mice. Error bars are standard deviations, and p-values refer to two-tailed T-test results.

In the bronchioles, the density of club and ciliated cells stayed constant over the lifespan: 149 ± 13 club cells and 96 ± 26 ciliated cells per mm basement membrane at 3 months and 153 ± 12 club cells and 91 ± 12 ciliated cells per mm basement membrane at 22 months (Fig. 4a-c). The proportion of EdU+ club cells fell from 17.7% ± 4.3% to 10.7% ± 3.7% with aging, and the proportion of EdU+ ciliated cells fell from 13.5% ± 3.5% to 8.6% ± 2.0% (Fig. 4a,b,d). It has previously been reported that club cells self-renew and differentiate to produce terminally differentiated ciliated cells^29^. Our data therefore indicate that in 22-month old mice, compared to 3-month old controls, there is a 40% lower club cell self-renewal rate. Our data also indicate a 36% lower rate of production of ciliated cells from club cells. As overall cell density in the bronchioles remained constant, this likely reflects an overall slowdown in cell turnover rates. An alternative explanation for the presence of EdU+ ciliated cells would be that ciliated cells themselves divide at homeostasis, and that the rate of their division falls ageing. Previous studies would not support this^12^, but we cannot exclude the possibility.

### Aging lung epithelial cells display changes in their gene expression profile

**Figure 5:**
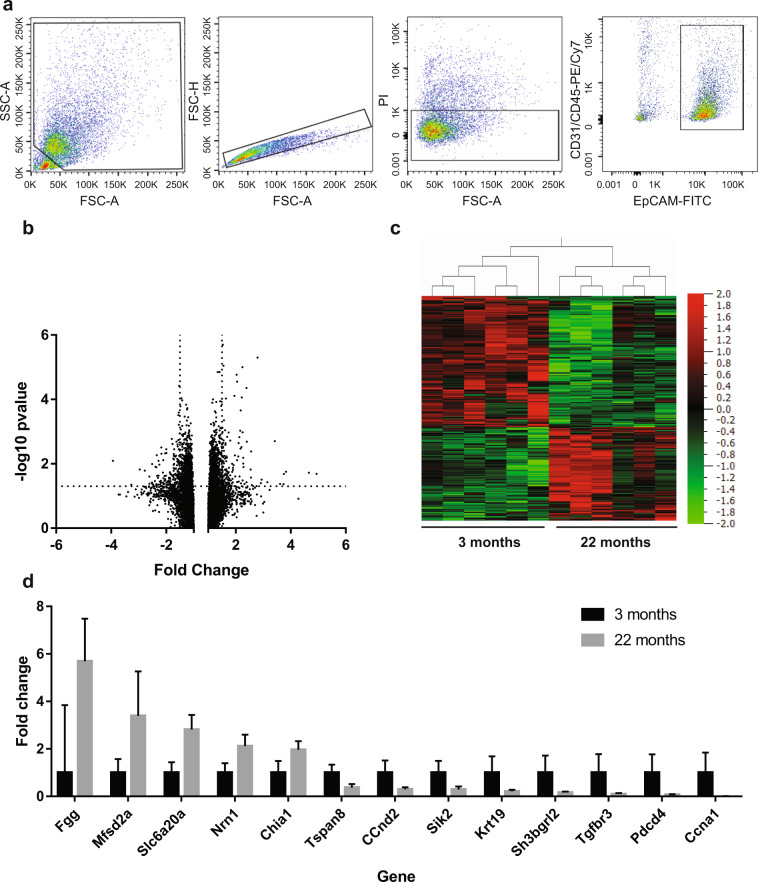
There are multiple age-related gene expression changes in lung epithelial cells. (**a**) Cell isolation strategy by FACS. CD31- CD45- EpCAM+ cells were sorted. (**b**) Volcano plot to show distribution of gene expression. Cutoffs of -log10 p-value > 1.3 and fold change > ±1.5 were applied (dotted lines). (**c**) Heatmap depicting clustering of samples from 3 month old (n = 6) and 22 month old (n = 6) lung epithelia according to gene expression profile. (**d**) RT-PCR confirmation of differential regulation of genes. Expression was normalised to UBC. Error bars are standard deviations. All genes depicted are significantly differentially expressed (p < 0.05, two-tailed T-test).

CD45- CD31- EpCAM+ epithelial cells were sorted from lungs of six 3- and six 22-month old mice (Fig. 5a). RNA was isolated and amplified, and microarray analysis carried out. Cutoffs of p < 0.05 and fold change >1.5 were applied (Full dataset supplied in Supplementary Table 1). This yielded 92 genes that were downregulated with aging and 56 genes that were upregulated (Fig. 5b). Samples clustered by gene expression pattern according to their age group (Fig. 5c).

Ingenuity Pathway Analysis revealed multiple pathways and regulators that are linked to directional gene expression patterns observed in this dataset (Tables 1 and 2). There was upregulation of a cell death pathway, and downregulation of multiple proliferation and viability pathways (Table 1). This reflects the fact that aging is a reversal of the growth processes co-opted by cancer cells. There was downregulation of cellular invasion and metastasis pathways, which may reflect alterations in cellular motility or extracellular matrix deposition with aging. Encephalomyelitis and myelination pathways were downregulated with aging. Whilst myelin is known for its role in oligodendrocytes, it is also found at high levels in the alveoli, as an important component of surfactant^30^. The downregulation of these pathways may reflect alterations in surfactant production with aging.

**Table 1:** Pathways altered with aging. Pathways were grouped into categories, and B-H multiple testing p-values assigned.

Class	Category of pathways altered	B-H p-values
Diseases & disorders	Connective tissue disorders	0.005-0.007
	Immunological disease	0.005-0.009
	Organismal injury & abnormalities	0.005-0.009
	Respiratory disease	0.005-0.010
	Cancer	0.005-0.010
	Developmental disorder	0.005-0.010
	Metabolic disease	0.005-0.010
	Haematological disease	0.005-0.010
	Skeletal & muscular disorders	0.005-0.010
	Neurological disease	0.005-0.010
	Endocrine system disorders	0.005-0.010
	Hereditary disorder	0.005-0.010
	Renal and urological disease	0.005-0.010
Development & function	Organ morphology	0.005-0.010
	Organismal development	0.005-0.010
	Haematological system development & function	0.005-0.010
	Nervous system development & function	0.005-0.010
Molecular & cellular mechanisms	Cellular assembly & organisation	0.005-0.008
	Cellular compromise	0.005-0.008
	Cell death & survival	0.005-0.010
	Cellular growth & proliferation	0.005-0.010
	Cell cycle	0.005-0.010
	Cell-to-cell signalling & interaction	0.005-0.010
	Cellular movement	0.005-0.010
	Cellular function & maintenance	0.005-0.010

**Table 2:** Regulators that may mediate age-related changes in gene expression.

Upstream Regulator	Activation Z-score	p-value
OSM	2.2	0.0001
Butyric acid	2.0	0.0099
CSF3	2.0	0.0140
THRB	2.0	0.0009
PP1	2.0	0.0001
Mir-16-5p	2.0	0.0234
PRDM1	1.6	0.0012
PS98059	1.6	0.0099
NFKBI	1.6	0.0094
IL6	1.5	0.0138
MYD88	−1.6	0.0027
TP53	−1.7	0.0393
ERK1/2	−1.8	0.0047
PPARA	−1.9	0.0021
β-estradiol	−2.0	0.0002
AKT1	−2.0	0.0003
MYB	−2.0	0.0038
FGF2	−2.2	0.0434
PI3K complex	−2.2	0.0009

Of the regulators (Table 2), only thyroid hormone receptor β (*Thrb*) is predicted to be activated. Thyroid signalling is necessary for postnatal lung septation and surfactant secretion, and thyroid treatment attenuates lung fibrosis^31,32^. Known regulator of airway cell differentiation, *Myb*,^33^ is predicted to be the most strongly inhibited, and indeed its expression was downregulated at the RNA level within the microarray experiment. Other upstream regulators predicted to be inhibited include *Tp53, Nfkbia, Prdm1* and *Creb1*, which regulate cellular activities such as proliferation, migration and inflammation in the context of lung cancer^34–37^.

Many of the regulators and pathways listed above centred on a module of common genes that were differentially expressed in our microarray data. To confirm their differential expression, we performed RT-PCR using commercially validated TaqMan assays on 96-well array plates (ThermoFisher, primer AssayIDs shown in Supplementary Table 2). 5 housekeeping genes were included (*18 S rRNA*, *Tbp, Ppia, Rab7* and *Ubc*) that are commonly used for normalisation of RT-PCR data from young adult mice. We checked whether the expression of these housekeeping genes changes with aging, by comparing their expression patterns each other. The Ct values of *Ppia* (p = 0.01) and *Rab7* (p = 0.04) were lower from the 22-month old samples than the 3-month old samples (Supplementary Fig. 3a). Notably, *Ppia* expression was significantly different to all other housekeeping genes (p = 0.05, Supplementary Fig. 3a). The expression of the remaining housekeepers, *18 S*, *Ubc* and *Tbp* tracked well with each other, and *Ubc* was selected as the gene to which the expression of test genes was normalised.

We confirmed age-related differential expression of 13 of candidate genes (Fig. 5d). In keeping with our EdU data indicating reduced proliferation rates in aging lung (Fig. 3d & 4d), there is marked downregulation of the proliferation-related genes *Pdcd4*, *Ccna1* and *CCnd2*. There is downregulation of *Tspan8*, which is involved in adhesion, and *Keratin 19*, which is implicated in epidermal aging^38^. There is downregulation of *Sik2* from the IGF pathway, which is well known to have a role in aging, and of *Tgfbr3*, from the TGFβ pathway, which is important for lung development and pathogenesis of pulmonary fibrosis. There is downregulation of the redox gene *Sh3bgrl2*, which is interesting as changes in redox homeostasis are a hallmark of aging. The antifungal enzyme *Chia* is upregulated, in keeping with a recent study showing accumulation of chitin in aging lungs and pulmonary fibrosis^39^. There is upregulation of extracellular glycoprotein *Fgg*, transport proteins *Mfsd2a* and *Slc6a20a*, and neural differentiation-associated gene, *Nrn1*.

## Discussion

In this study, we asked how lung epithelial progenitor activities change with aging, and how this correlates with changes in gene expression profile and overall tissue architecture. Our aim was to understand the baseline changes that occur with healthy aging over the natural lifespan. Aging is thought to directly contribute to the pathogenesis of IPF, COPD and lung cancer along with multiple other factors. Our data will help to disentangle the specific contribution of aging to disease pathogenesis.

We carried out our morphometric studies using high-throughput, automated analysis of whole H&E stained sections to eliminate investigator bias. Previous studies have demonstrated age-related airspace enlargement by measuring an increase in the mean linear intercept, mean alveolar volume, or ductal airspace volume^2–5,40,41^. We could not confirm this at a statistically significant level, although there was a trend towards increased mean linear intercept, increased airspace area and reduced alveolar parenchymal tissue density with age (Fig. 1a-e). We also identified that bronchioles were smaller in terms of their total cross-sectional area and diameter, and that alveolar wall area was also reduced (Fig. 2a,b,d,f, Supp Fig. 1). These are novel and very interesting findings with two possible explanations. Firstly, alveolar expansion with aging could lead to a reduction of alveolar tethering to bronchioles and thus a failure to maintain bronchiolar cross-dimensional area. Secondly, there may be increased bronchoconstriction with age. The presence of increased bronchoconstriction in elderly patients has been inferred by measuring lung function after administration of an agonist of the parasympathetic nervous system^42^. Our observation of reduced bronchiolar cross-sectional area at the morphological level is an important development, and may have clinical implications, for example in the administration of bronchodilators to the elderly.

We studied the density and proliferative activities of distal lung progenitors (bronchiolar club and alveolar Type II cells) in young and old mice, by long-term EdU administration and immunohistochemistry. We found that the density of club cells and ciliated cells present in the bronchioles remained constant with aging (Fig. 4a-c). This is in contrast to our and others’ previous data from the trachea which demonstrated a reduction in cellular density with aging^12,13^. However, the cellular composition of the tracheal epithelium is different as it contains basal cells in addition to club and ciliated cells, whereas the murine distal airways do not. Although the cellular composition of the epithelium remains stable with aging, we found that rates of club cell self-renewal and production of EdU+ ciliated cells fell by 40% and 36%, respectively (Fig. 4a,b,d). The reduced club cell proliferation rate that we observed was in agreement with a previous study which found that the percentage of PCNA + proliferating cells fell by 69% between 2 and 24 months of age^43^. Notably, the baseline level of PCNA + cells in young mice in this study was 0.068%, whereas our baseline level of EdU incorporation over 2 weeks in club cells in young mice was 17.7%. The larger window of our readout may facilitate determination of the extent of the age-related decrease. Maintenance of the cellular composition of the epithelium in the presence of a reduced cellular proliferation rate points to an overall slowdown in bronchiolar epithelial cell turnover. It also suggests a decreased capacity for regeneration with aging, which could contribute to the pathogenesis of chronic lung disease and increased susceptibility to infection. This needs to be tested in damage models, however, to exclude the possibility that the proliferative capacity of bronchiolar cells could be restored in response to damage.

To quantify alveolar cell number in a manner that was not confounded by alterations in gross alveolar structure, counts of Type II and Type I cells were normalised to tissue area, resulting in alveolar cell density measurements. We note that a previous study argued that stereology may provide a more accurate assessment of changes in alveolar Type II cell number than planimetric approaches, or indeed by FACS^44^. Our previous studies nonetheless showed that automated planimetric histological analysis can provide accurate and detailed descriptions of changes in lung structure in different injury models^24,25^. We are confident therefore that our study provides interesting and novel insights into lung progenitor dynamics with aging. Quantifying the cells according to our methodology, we found no changes in the density of Type II cells with aging (Fig. 3a-c). This is in keeping with a recent paper which demonstrated a constant ratio of Type II cells per alveolus with aging^41^. Notably, however, the density of Type I cells was reduced by 34% with aging (Fig. 3a-c). Previous studies have shown that Type II cell proliferation and differentiation into Type I cells occurs at a low level at homeostasis, and is maintained or elevated in certain injury-repair models^14,27^. In our study we showed that with aging, Type II cell self-renewal was maintained, whereas production of EdU+ Type I cells was reduced by half (Fig. 3a,b,d). It is interesting and somewhat unexpected that Type II cell numbers and self-renewal rates do not change. The inferred reduced Type II to Type I cell differentiation rate may represent a decline in progenitor function, and is reminiscent of analogous situations in the brain and skin in which progenitor number is maintained with aging, but function is not^45,46^. An inhibited Type II cell differentiation capacity may contribute to the pathogenesis of chronic lung disease. An alternative explanation for the inferred reduced Type II-Type I cell differentiation rate is that Type I cell turnover rate may fall with aging. Lineage tracing of Type II and Type I cells in young and old mice would be required to resolve this. In addition, we note that we made the assumption that Type I cells are terminally differentiated and do not proliferate or dedifferentiate at homeostasis, in contrast to their behaviour under certain injury conditions^47^. We cannot however exclude the possibility that such processes do occur and contribute to the observed dynamics.

Type II cell heterogeneity has been demonstrated in recent studies, which showed the existence of a rare Axin2+ subpopulation with greater clonogenicity than bulk Type II cells^48–50^. We note that as EdU incorporation analysis is an unbiased approach, it will capture the overall behaviour of all Type II cell subpopulations, amongst which bulk Type II cells predominate. Our study does not therefore address whether these Type II cell subpopulations undergo differential changes with aging, and this would be an interesting topic of future study. Furthermore, it is unclear whether reduction in Type II to Type I cell differentiation drives airspace enlargement and reduced alveolar tissue density, or whether it occurs secondarily to changes in the stromal compartment. An important area of future research is to understand how the epithelial, fibroblast, endothelial, and immune cell compartments interact with each other to effect cellular and morphological changes in the aging lung.

We determined the changes in gene expression that occur in the lung epithelium with aging by sorting epithelial cells and performing microarray analysis. Pathway analysis identified changes in several pathways associated with proliferation, cell death and cellular motility, suggesting that these processes are modulated by aging. We noted down-regulation of multiple genes associated with proliferation, in keeping with our EdU data. One limitation of our study is that gene expression was measured in bulk across all EpCAM+ epithelial cells, so changes in expression in individual epithelial cell populations are not accounted for, nor is the heterogeneity of gene expression within these populations. Different epithelial cell types have different metabolic and proliferative profiles, and these will be obscured in our analysis as they are studied in bulk.

A recent study avoided such limitations by applying single cell RNA-seq to lung cell populations, alongside proteomics^51^. This study found complex global changes in the aging lungs, including increased transcriptional noise indicative of deregulated epigenetic control and extracellular matrix remodelling. It also found cell-type specific changes, including an increase in mTOR signalling in Type II and club cells, in keeping with our observation of decreased epithelial expression of Sik2, a negative regulator of this pathway (Fig. 5d). It also found that cholesterol biosynthesis was increased in aged Type II cells, in keeping with our observation that myelin biosynthesis pathways were altered (Table 1). Interestingly, bulk RNA-seq data from that study suggested that there was an increase in expression of ciliated cell markers, which the authors interpreted as an increase in ciliated cell number. This was not borne out by our immunohistochemistry analysis (Fig. 4a-c). We note that an alternative explanation for their data is the increased expression of ciliated cell markers within individual cells. An important focus of future work will be test the functional importance of observed genetic changes in the aging lung, as these genes may represent novel druggable targets for reversing the effects of aging and treating chronic lung disease. A recent study carried out such functional work. It showed that Wnt signalling activated in Type II cells from old mice, and demonstrated that activation of Wnt signalling in primary Type II cells could recapitulate age-associated senescence^52^. Similar studies are required looking at the other pathways that change with aging.

The changes that we have observed in our experimentally tractable mouse system will provide a helpful basis for studying these features in humans. It will be important to determine if similar cellular and molecular mechanisms operate in the human lung. Given that the human lifespan is far longer than the rodent lifespan, it will be interesting to determine whether age-related changes in progenitor function are indeed comparable to mice, or whether they are in fact more pronounced, due to the longer timescales involved. Additionally, stem cell exhaustion is a key hallmark of aging, but occurs in the context of multiple other changes, such as epigenetic alterations, mitochondrial dysfunction, loss of proteostasis. The specific contribution of each of these variables to lung aging, and in turn to age-related lung disease, will need to be carefully examined. A greater understanding of lung aging will contribute to development of more effective therapies for chronic lung disease in the future.

## Methods

### Mouse husbandry and EdU treatment

All animal experiments were performed in accordance with the Animals (Scientific Procedures) Act 1986 Amendment Regulations 2012 following ethical approval by the University of Cambridge Animal Welfare and Ethical Review Body (AWERB) (license number PPL70/812). C57/B6J mice were used. Mice were fed expanded breeding chow (RM3E, Special Diet Services), were screened rigorously for infection, and were not provided with exercise equipment. For EdU incorporation experiments, mice were administered EdU (0.3 mg/ml) + sucrose (2%) in drinking water ad libitum for 14 days prior to culling them.

### Tissue processing

Mice were culled by injection of a lethal dose of Euthatal. Lungs were inflated manually until they reached their maximal volume with paraformaldehyde (4%) and then submerged in paraformaldehyde (4%) for 4 hours at 4°C. They were rinsed three times, then washed three times for 15 minutes each in PBS. They were incubated in 15% sucrose, 20% sucrose, 30% sucrose for an hour each at room temperature. They were incubated in a 1:1 mixture of 30% sucrose: OCT overnight, and then embedded in OCT. Lung lobes were embedded separately and all experiments were carried out using only the left lung. Blocks were serially sectioned in their entirety, with three 10 µm tissue sections per slide, generating approximately 100 slides per block.

### Immunohistochemistry

For H&E analysis, every tenth slide from each block was stained using standard methods. For immunofluorescent staining, every twentieth slide from each block was stained. Primary antibodies were acetylated tubulin (mouse, 1:1000, Sigma, T7451), E-cadherin (rat, 1:1000, ThermoFisher, 13-1900), Hopx (rabbit, 1:50, Santa Cruz, sc-30216, clone FL-73), and LAMP3 (rat, 1:100, Dendritics, DDX0192, clone 1006F7.05). For Hopx staining, antigen retrieval was achieved by boiling in 10 mM sodium citrate + 0.05% Tween-20, pH6 for 15 minutes, and allowing to cool to room temperature over 45 minutes. EdU was detected using the Click-It EdU AlexaFluor-555 Imaging Kit (C10338, ThermoFisher), after secondary antibody treatment. AlexaFluor conjugated secondary antibodies (ThermoFisher) were used: donkey anti-rabbit 488 (A10040), donkey anti-rat 488 (A21208), goat anti-rat 647 (A21247) and donkey anti-mouse 647 (A31571).

For the analysis of alveolar stainings, >3 representative images were taken of each slide. For the analysis of bronchiolar stainings, 1-8 images were taken per slide, depending on the frequency of bronchioles present on the slide. For analysis of alveolar stainings, Type II cells were identified as cells with Lamp3+ vesicles throughout the cytoplasm, and Type I cells were identified as cells with Hopx+ nuclei. For analysis of bronchiolar stainings, individual cells were delineated by lateral E-cadherin staining. These cells were then identified as either ciliated cells by apical acetylated tubulin staining, or as club cells by cytoplasmic CC10 staining. EdU+ cells were identified as those with EdU+ nuclei. The position of the basement membrane was inferred by drawing a line between the basalmost points of the lateral E-cadherin staining, and this was then used to determine the density of bronchiolar cells.

Cell counting was carried out manually using ImageJ’s “cell counter” plug-in. Cells of each type were marked off as they were counted, to ensure accuracy. For the analysis of alveolar images, >950 cells were counted for each biological replicate, and for the analysis of images of bronchioles, >200 cells were counted for each biological replicate. The inferred position of the basement membrane was measure by drawing a line on ImageJ using the “freehand line” feature, and measuring its length.

### Microscopy and automated image analysis

H&E stained slides were scanned on an Axio Scan Z.1 (Zeiss, Germany) and digital images of full lung sections were recorded at x20 magnification with a pixel size of 0.452 µm. Fully automated quantification of morphological changes of lung tissue was carried out by Biocellvia (Marseille, France) using their validated proprietary software programs dedicated to respiratory diseases^24,25^. Dedicated algorithms were developed to automatically discriminate bronchioles of which the Feret diameter (the largest diameter) ranged between 100 and 500 µm. Then, automated delineation of the inner and outer limits of the bronchiolar epithelium was carried out, in order to delineate the lumen and the basal membrane (Supp Fig. 1a-c). The mean value of key morphometric parameters of bronchioles were assessed: the bronchiolar, lumen and wall area, the bronchiolar and lumen diameter (Feret diameter) and the wall thickness (Supp Fig. 1d). The wall thickness corresponds to the shorter distance between two pixels located at the outer and inner epithelium limits, whose average value covers several thousand measurements. Biocellvia’s programs were also used to analyse morphometric changes occurring in the pulmonary airspaces, on the same lung images in which large bronchi and vessels were automatically removed. Three key morphometric parameters were assessed: the airspace area (area of airspaces/total area of lung section), the alveolar tissue density (alveolar tissue area/total area of lung section) and the mean linear intercept (Lm) (Supp Fig. 1e). The assessment of Lm we developed in the present study was not calculated by counting the number of intersections with the alveolar walls and dividing it by the total length of the grid lines. Our measurement of the distance between alveolar walls corresponded to the mean Feret diameter (largest diameter) of the closed airspaces^24^. In contrast to the conventional Lm measure, which is performed on few manually selected regions of interest, the measure of Feret diameter was performed on the whole closed airspaces contained in the entire lung section. In addition, as for airspace area and tissue density, the Lm measure was fully automatic and totally independent of the experimenter.

For alveolar tissue density analysis, >8 images were analysed per biological replicate. For alveolar area, >2 images were analysed per biological replicate. Each image was of a section of the left lung, sectioned longitudinally. Alveolar area was automatically determined, and mean values generated. For bronchiolar analysis,>7 individual images of bronchioles were manually selected and then automatically analysed for each biological replicate. Immunoflourescent slides were imaged on an Olympus microscope or a Leica SP5, where specified. 3-5 fields of view were imaged per slide. Cells were counted manually in Fiji (ImageJ, NIH).

For statistical analysis of morphometric and cell density data, all technical replicates were combined to generate mean measurements for each biological replicate. Using these biological means, two-tailed T-tests were then carried out.

### Epithelial cell isolation

Lungs were perfused with PBS via the right ventricle. They were finely chopped and digested using dispase (10U/ml, Roche-4942086001, Sigma) for 30 minutes at room temperature, and then DNase (0.33U/ml, Roche-10104159001, Sigma) for 10 minutes at room temperature. DMEM + FBS (10%) was added and cells were passed through a 0.5 mm wire mesh (tea strainer) and then a 100 µm filter. Cells were pelleted and resuspended in ACK red blood cell lysis buffer (Invitrogen) for 5 minutes and washed in PBS + BSA (3%). Cells were resuspended at 10 million cells/ml and incubated with biotinylated anti-CD45 (1:250, 103103, Biolegend) and biotinylated anti-CD31 (1:250, 558737, BD) for 20 minutes on ice, and washed in PBS + BSA (3%). 5µl Dynabeads (11047, ThermoFisher) per million cells were washed twice in 10 ml PBS, and mixed with cells. Cells were loaded onto a magnet for 3 minutes, CD31-CD45- cells were poured off, and this step repeated. Cells were then incubated with FITC-conjugated anti-EpCAM (1:200, 118208, Biolegend) + Streptavidin-PE-Cy7 (1:200, 405206, Biolegend) + DAPI (1:5000), and washed in PBS + BSA (3%). Remaining contaminant CD31/45+ cells were excluded, and EpCAM+ singlet cells were sorted on an Aria cell sorter.

### RNA isolation, microarray analysis and RT-PCR confirmation

RNA was isolated from lung epithelial cells immediately after they were isolated, using the MirVana kit (AM1560, ThermoFisher). RNA quantity and quality was checked on a Nanodrop 1000 (ThermoFisher) and a BioAnalyzer (Agilent), respectively. RNA was amplified and biotinylated sense strand cDNA generated using the GeneChip WT Pico Reagent Kit (Affymetrix). cDNA was hybridised to GeneChip Mouse Transcriptome Assay 1.10 microarrays, using a GeneChip Fluidics Station (Affymetrix). Arrays were scanned using a GeneChip Scanner system (Affymetrix). Microarray data quality control was carried out using Qlucore software. Differential expression values were generated using the Affymetrix Transcriptome Analysis Console, and Ingenuity Pathway Analysis (Mar 2018 Release) was used for further data analysis. Following microarray analysis, differential expression of genes was confirmed using custom TaqMan Array Cards (ThermoFisher).

## Supplementary information

Supplementary Information.

Supplementary table 1

Supplementary table 2
