# Supplementary material for: Distal lung epithelial progenitor cell function declines with age
Source: Sci Rep. 2020 Jun 26;10:10490. doi: 10.1038/s41598-020-66966-y (PMC7319976; doi:10.1038/s41598-020-66966-y)
Supplement: Supplementary file 1 — Supplementary Information. [file 41598_2020_66966_MOESM1_ESM.pdf]

Supplementary information

Distal lung epithelial progenitor cell function declines with age

Julie K. Watson, Philip Sanders, Rebecca Dunmore, Guglielmo Rosignoli, Yvon Julé, Emma L. Rawlins, Tomas Mustelin, Richard May, Deborah Clarke, Donna K. Finch.

Supplementary Figure 1

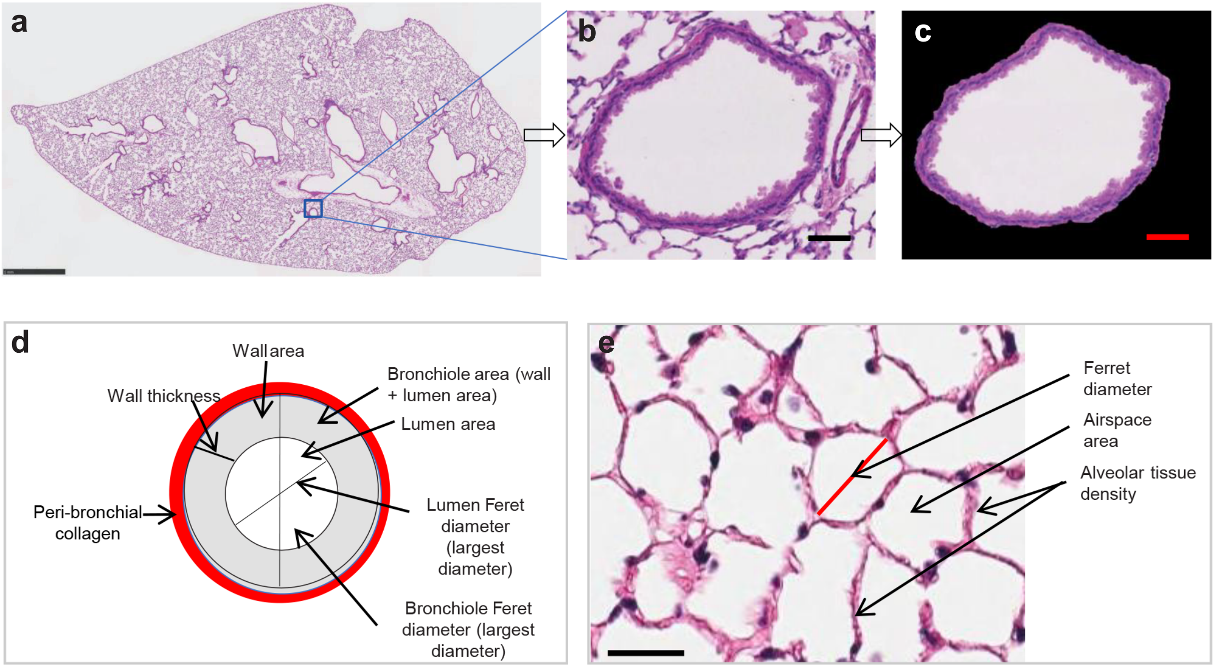

Supplementary Figure 2

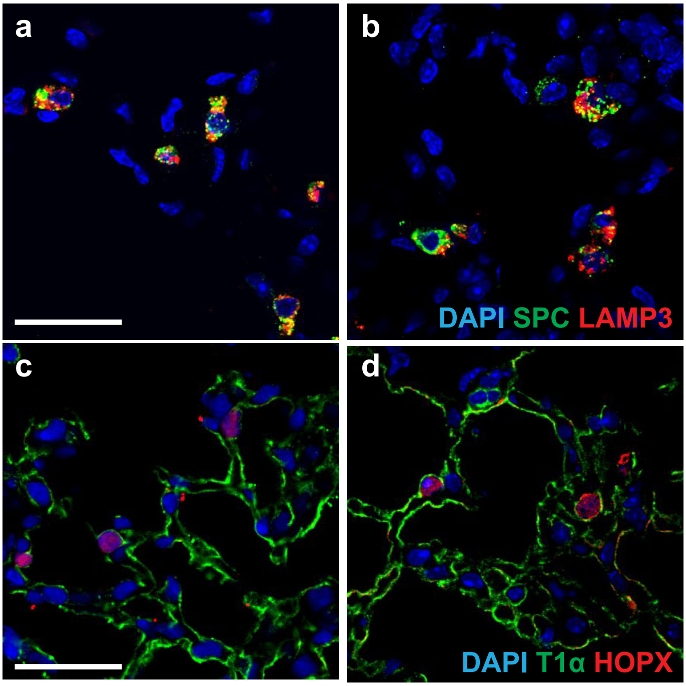

Supplementary Figure 3

a

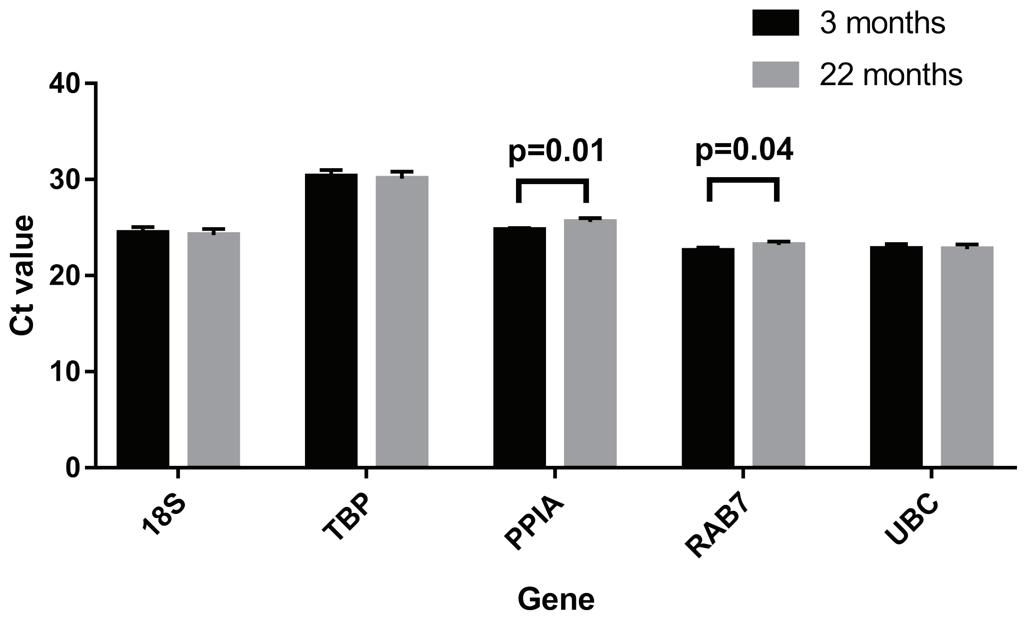

b

		Gene to which expression is normalised				
		18S	PPIA	RAB7	TBP	UBC
Fold change (p-value)	18S		1.8 (0.04)*	1.2 (0.67)	0.8 (0.59)	1.0 (0.91)
	PPIA	0.4 (0.02)*		1.2 (0.01)*	0.4 (0.15)	0.5 (0.02)*
	RAB7	0.4 (0.06)	1.1 (0.24)		0.5 (0.25)	0.6 (0.05)*
	TBP	0.8 (0.54)	1.9 (0.03)*	0.6 (0.30)		1.2 (0.59)
	UBC	0.8 (0.41)	1.6 (0.05)*	1.0 (0.91)	0.9 (0.73)	

Supplementary Figure Legends

Supplementary Figure 1. Schematic depicting the automatic analysis of bronchioles and airspaces from an entire lung section. a. Bronchioles are first selected according to their Feret diameter (largest diameter) ranging between 100 and 500 µm. b, c. Bronchiolar epithelium is then automatically discriminated by removing the attached alveolar tissue. d. Morphological parameters are assessed including: bronchiolar, lumen and wall area, the bronchiolar and lumen Feret diameter and the wall thickness. e. Representative image of airspace morphometric parameters assessed for alveolar tissue analysis. Scale bars are 1 mm (a), 50 µm (b, c), 30 µm (e).

Supplementary Figure 2. Validation of markers of Type II and Type I cells. 63x images of lung alveoli from a. 3 month old and b. 22 month old mice, stained for DAPI (blue), SPC (green), LAMP3 (red), and from c. 3 month old and d. 22 month old mice, stained for DAPI (blue), T1α (green) Hopx (red). Scale bars are 15µm.

Supplementary Figure 3. Expression of housekeeping genes. a. Raw Ct values for each housekeeping gene. Error bars are 95% confidence intervals. n=6 mice per age group. b. Table demonstrating the fold change of each housekeeping gene, with respect to the other housekeeping genes, and the accompanying two-tailed T-test p-value. P-values < 0.05 are marked by an asterisk.
